# A Complication of an Endoscopic Pigtail Stent Migration into the Cavity during Deployment as a Treatment for Gastric Leak

**DOI:** 10.1155/2019/6974527

**Published:** 2019-09-10

**Authors:** Abrar A. AlAtwan, Ali AlJewaied, Talal AlKhadher, Mohannad AlHaddad, Iqbal Siddique

**Affiliations:** ^1^Faculty of Medicine, Kuwait University, Jabriya, Kuwait; ^2^Department of Surgery, Al Amiri Hospital, Kuwait City, Kuwait; ^3^Thunayan Alghanim, Center of Gastroenterology, Amiri Hospital, Kuwait City, Kuwait

## Abstract

Gastric leak following gastrointestinal surgery is the most dreadful complication, which implies long hospital stay, morbidities, and not irrelevant mortalities. There is no standard recommendation for treating postlaparoscopic sleeve gastrectomy leak, which makes its management challenging. Endoscopic internal drainage by double-pigtail drains currently became the recommended approach. Complications to this approach include bleeding, ulceration at the tip of the double-pigtail stent, and uncommon migration. Here, we report our experience with drain displacement into the cavity while deployment in a patient who experienced gastric leakage after undergoing sleeve gastrectomy.

## 1. Introduction

Staple line leak postlaparoscopic sleeve gastrectomy is one of the most serious and life-threatening complications and is reported to occur at an average rate of 2.8 ± 2.6% (range 0-8%) [1]. Currently, no specific standardized approach is established, but adequate draining and gastric decompression are the main goals of treatment for leaks. Frequently, Self-Expandable Metal Stents (SEMS) is used for the treatment of postsurgical gastric leak due to research findings indicating it is associated with high success rates. Endoscopic internal drainage (EID) by means of a double-pigtail stent is increasingly used as an effective approach after studies showed it required fewer procedures per patient, was better tolerated, and had lower morbidity-mortality compared to SEMS [2, 3]. However, this technique is not free of complications and physicians should be aware of the possible complications.

## 2. Case Presentation

**Figure 1:**
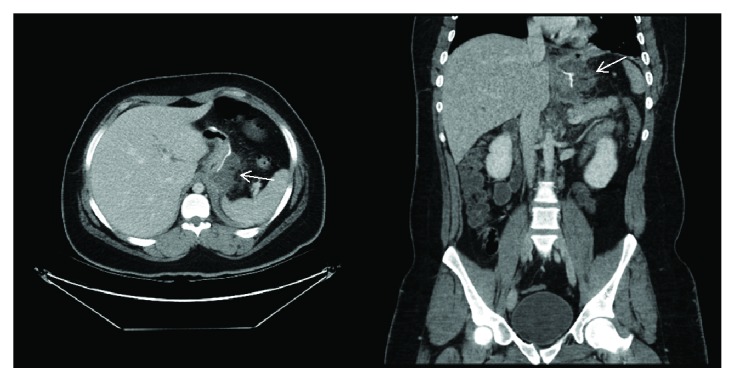
Computed tomographic scan showing perigastric collection adjacent to gastroesophageal junction and along sleeve gastrectomy sutures. Arrow: perigastric collection.

**Figure 2:**
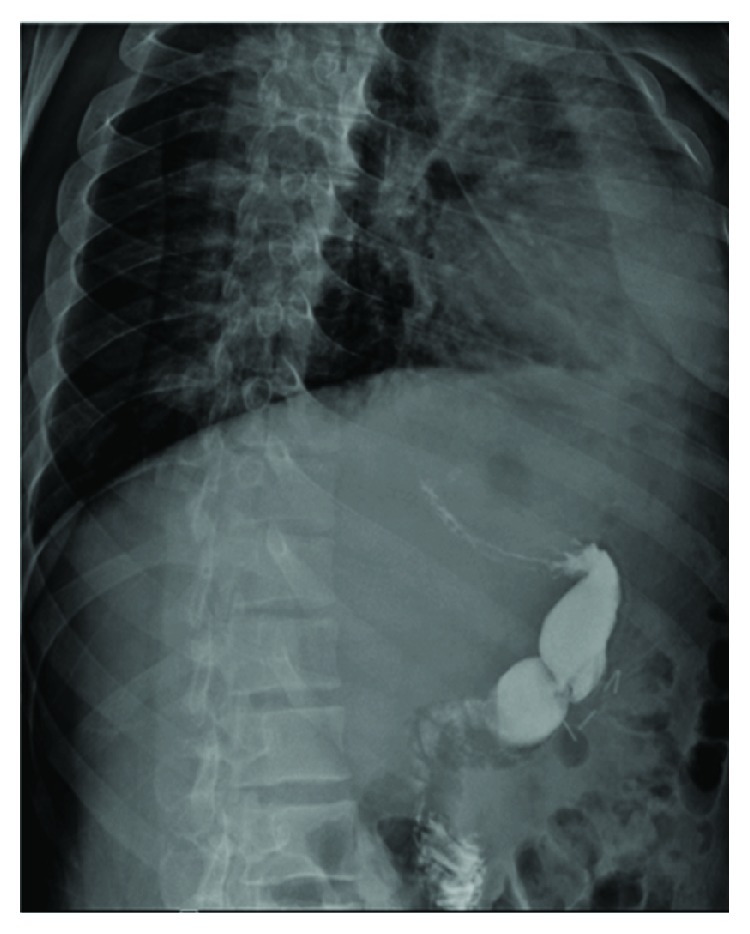
Upper gastrointestinal series with Gastrografin swallow showing no leakage.

A 22-year-old man weighing 115 kg and with a body mass index (BMI) of 40 kg/m^2^ underwent laparoscopic sleeve gastrectomy at an outside institution on 18/8/2018 for the treatment of morbid obesity. At postoperative day 21, he presented with epigastric pain, nausea, and fever. The abdomen was tender without signs of peritonitis. His lab result showed WBC of 31.4 × 10^9^/L and a procalcitonin level of 0.64 ng/mL. CT abdomen was done and revealed 2 epigastric collections 3 × 4 × 4 cm and 6 × 5 × 4 cm likely to be an early abscess (Figure 1). A Gastrografin study was also done and showed no leak (Figure 2). Radiological drainage was unsuccessful due to the size of the left liver lobe and the superimposed bowel loops.

On 13/9/2018, the patient deteriorated and was taken to the operation theatre for diagnostic laparoscopy. Gross adhesions were encountered, and dissection of the omentum was difficult to perform due to distorted anatomy and the fear of injuring the splenic artery. Therefore, a drain was fixed just behind the spleen and the surgery was aborted.

**Figure 3:**
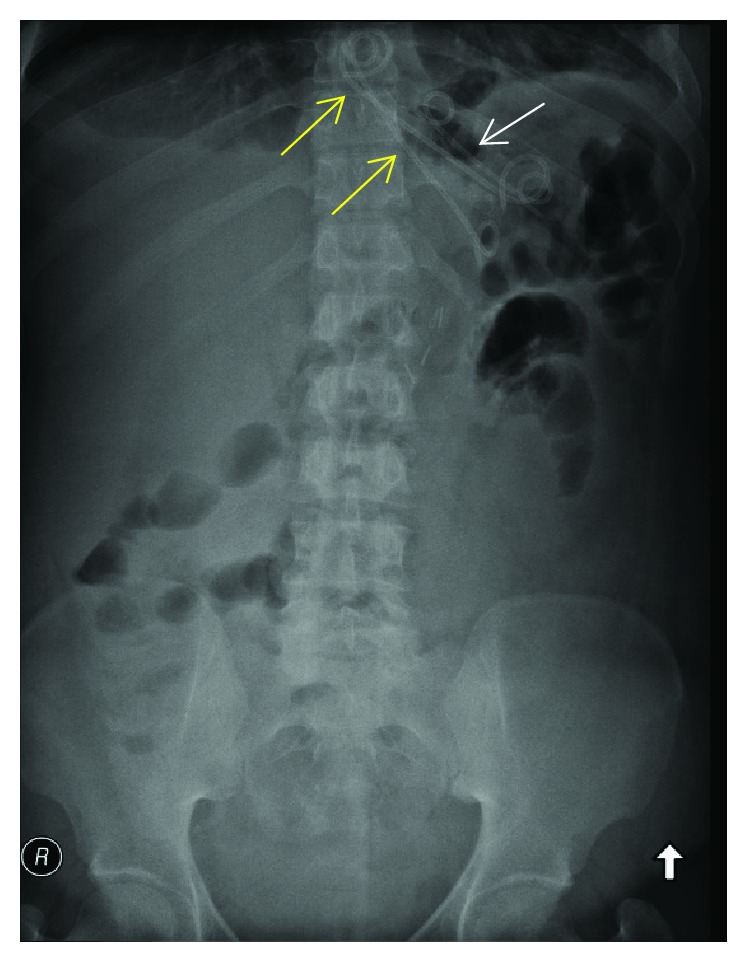
X-ray abdomen showing the displacement of one double-pigtail drain into the collection (white arrow). A satisfying double-pigtail drain position across the leak orifice (yellow arrows).

**Figure 4:**
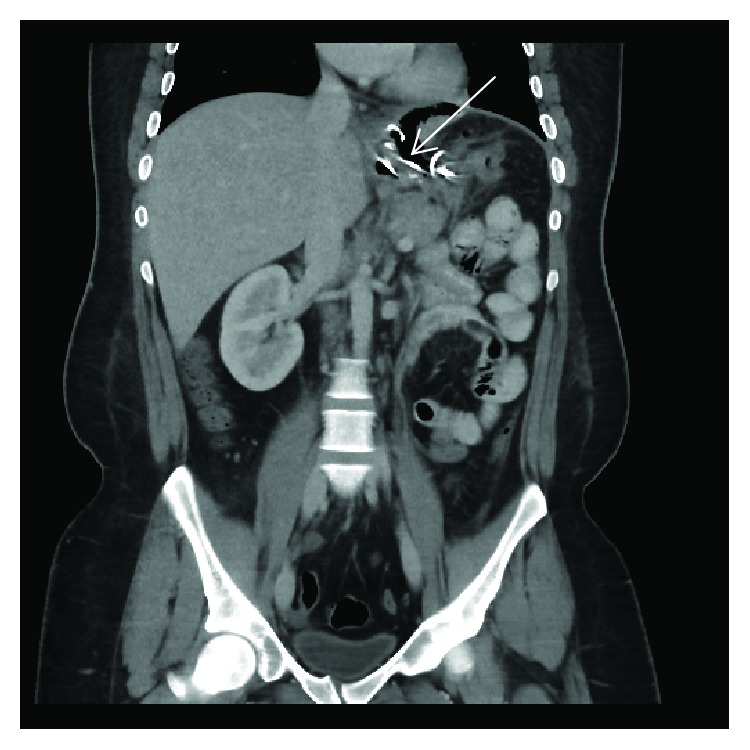
Abdominal computed tomographic scan with intravenous contrast shows no leakage, and as compared to the previous study, the collection regressed after putting a stent. Arrow: displaced stent into the collection.

An upper gastrointestinal endoscopy was performed on 20/9/2018 during which a fistulous opening 40 cm from the incisure just distal to the gastroesophageal junction was cannulated with an ERCP cannula. Contrast was injected and the leak was confirmed. A guidewire was then passed into the cavity. A double-pigtail stent was initially placed through the fistula into the cavity; however, during deployment, the stent migrated completely into the cavity (Figure 3). Attempts to remove the stent with forceps or snare were not successful. Two pigtail stents 7 cm and 10 cm were then placed in the cavity. The case was discussed with various bariatric surgeons regarding the foreign body, and all advised to watch and wait and to retrieve the foreign body during endoscopy when the space shrinks. On 30/9/2018, a repeat CT was done and revealed no evidence of oral water-soluble contrast leak, and it also showed significant improvement in the fluid collection (Figure 4).

On 8/10/2018, the patient complained of left upper quadrant pain associated with nausea and vomiting. The patient was booked for upper GI endoscopy that showed the previously placed stents in the esophagus; therefore, they were removed. The fistulous opening was cannulated with an ERCP cannula, contrast was injected, and the leak was confirmed. A pair of forceps was passed through the fistula into the cavity, and the migrated stent was grabbed and pulled out completely. Two 7Fr 5 cm double-pigtail stents were then placed in the cavity. The patient had an uneventful recovery thereafter with no further complications and no complaints.

## 3. Discussion

Gastric leaks after laparoscopic sleeve gastrectomy are the most dreadful complication due to their associated high morbidity and mortality. After pulmonary embolism, they represent the second most common complication leading to death after bariatric surgery [4]. The pathogenesis of leaks is believed to be due to local ischemia near the staple line from the use of electrocautery in combination with higher intraluminal pressure postoperatively, rather than staple line dehiscence [5].

Management of post-LSG gastric leaks is challenging since a standardized approach has not yet been established. Therefore, the management plan depends on the time of diagnosis and size of the leak and on the clinical presentation. Many conservative modalities are available for leaks post-LSG including surgical [6, 7], endoscopic [8], or percutaneous drainage [9]. The most common treatment is with deployment of SEMS, but the use of SEMS is burdened with a high migration rate and poor tolerance [8, 10].

Nowadays, endoscopic internal drainage (EID) by means of a double-pigtail stent is commonly used as a first-line approach. In this approach, 1 or 2 double-pigtail drains are placed through the leak with one end in the collection and the other in the remnant stomach and they are exchanged regularly over 4-6 weeks until complete fistula healing. This technique is aimed at internally draining the perianastomotic fluid collection and at the same time promoting leak healing. Even though endoscopic internal drainage requires multiple endoscopic sessions, it is very effective and well tolerated. It also has fewer complications and is less expensive than stents [3, 11].

Complications related to this technique include GI ulceration at the tip of the double-pigtail stent and bleeding [3]. Very few cases of pigtail stent migration are reported in the literature, in which it was reported three times invading the spleen and once into the abdominal wall [12–15]. To our knowledge, this is the first case report of complete migration of a pigtail stent into the cavity during deployment, following endoscopic internal drainage. Since leaks or fistulas following sleeve gastrectomy occur at the proximal third of the stomach at the level of the cardiac notch in approximately 75%-87.5% [16, 17], they are in close proximity to the splenic parenchyma and splenic vessels, and therefore, there is a high possibility to injure these structures during maneuvers to deploy the pigtail stent [12]. In addition, the drain in the collection will act as a foreign body and might impede its closure if not retrieved. This complication should be well recognized and considered.
